# Effects of Topoisomerase II alpha Inhibition on Oral Cancer Cell Metabolism and Cancer Stem Cell Function

**DOI:** 10.26502/droh.0076

**Published:** 2024-04-29

**Authors:** Thanigaivelan Kanagasabai, Mariam Hawaz, Kayla Ellis, Orlyne Fah, Helana Mikhaeil, Philip Nguyen, Nathalie Tombo, Anil Shanker, Chethan Sampath, Zaid H Khoury, James Cade, Alexys Ferguson, Pandu Gangula

**Affiliations:** 1 Department of Biomedical Sciences, School of Graduate Studies, Meharry Medical College, Nashville, TN 37208, USA; 2 Department of Oral Diagnostic Sciences and Research, School of Dentistry, Meharry Medical College, Nashville, TN, 37208, USA; 3 Department of Biochemistry, Cancer Biology, Neuroscience and Pharmacology, School of Medicine, Meharry Medical College, Nashville, TN 37208, USA

**Keywords:** Oral cancer, Etoposide, Topoisomerase 2 alpha, Cancer stem cell marker, Cell proliferation, Cell metabolism, Apoptosis, Periodontitis

## Abstract

**Background:**

Topoisomerase IIα (TOP2A), is an enzyme involved in DNA replication, transcription, recombination, and chromatin remodeling and is found in a variety of cancers. However, the role of TOP2A regulation in oral cancer progression is not fully explained. We investigated the effect of TOP2A inhibition on cell survival, metabolism, and cancer stem cell self-renewal function in oral cancer cells.

**Methods:**

Oral carcinoma cell line SCC25 was cultured in complete DMEM/F12 media and treated with 5μM of Etoposide (Topoisomerase II inhibitor) for 48h. The critical parameters of cellular metabolism, including extracellular acidification rate (ECAR) and mitochondrial oxidative phosphorylation based on the oxygen consumption rate of cancer cells were assessed using Seahorse assay. Western blotting was performed to assess the proteins that are associated with proliferation (Survivin, IL-6) and cancer stem cell function (Oct4, Sox2) in cell lysates prepared from control and etoposide treated groups. Statistical analysis was performed using One-way ANOVA with Dunnett’s multiple comparisons test.

**Results:**

The protein expression of TOP2A was significantly (P<0.05) inhibited by etoposide. Additionally, TOP2A inhibition decreased the mitochondrial respiratory parameters including basal respiration, maximal respiration and ATP production. However, TOP2A inhibition has no impact on glycolytic function. Moreover, the proliferative marker survivin and IL-6 showed a significant (P<0.05) decrease after TOP2A inhibition. Conversely, the protein expression of cancer stem cell markers Oct-4 and Sox 2 were not altered.

**Conclusion:**

These results indicate that inhibition of TOP2A is more efficacious by decreasing the mitochondrial metabolic reprogramming and thereby downregulating the key anti-apoptotic and pro-survival mediators. Thus, TOP2A represents an ideal therapeutic target and offers a potential treatment strategy for OSCC.

## 1. Introduction

Oral squamous cell carcinoma (OSCC) is a prevalent and invasive head and neck cancer that originates from the surface epithelium of the oral cavity, with an overall 5-year survival rate of ~67% [1]. The increasing prevalence of OSCC contributes substantially to the overall burden of cancer-related health issues including related to swallowing, speech, and taste, resulting in significant impact on patient’s quality of life [2,3]. Indeed, lip, oral and pharyngeal cancers accounted for ~500,000 cases and 300,000 deaths in 2019 contributing to ~4.3 million disability-adjusted life years (DALYs) worldwide [4]. Even with the enhanced awareness and accessibility of healthcare to people worldwide, most oral and oropharyngeal cancers present at an advanced clinical stage and have morbid therapeutic outcomes [5]. In addition, recent studies have evidenced the strong correlation between oral cancer and the severity of periodontal disease. However, the molecular mechanism of this positive association is not well understood [6].

Despite the advancements of current treatment modalities of OSCC such as surgery, chemoradiation, and immunotherapy, successful treatment of advanced OSCC is an unmet clinical need [7]. This may be due to the resistant nature of OSCC to anticancer drugs through a wide variety of cellular and molecular mechanisms, including epigenetic changes, and genetic mutations [8]. In addition, genomic instability is one of the most important factors that lead to cancer development [9,10].

Aggressive malignancies are characterized by relatively uncontrolled cell proliferation making them especially dependent on topoisomerase enzymes to enable high rates of DNA replication and transcription [11]. DNA topoisomerase II (TOP2) is a ubiquitous nuclear enzyme that acts as a homodimer that catalyzes cleavage and re-ligation of DNA double strands breaks that allow the passage of the intact DNA segment. TOP2 genes have two genetically distinct isoforms, *TOP2A* and *TOP2B* with ~70% similar structural features [12].Topoisomerase II alpha (TOP2A) is a central regulators of chromatin topology that controls and maintain genomic integrity in various dynamic processes such as transcription, replication, and cell division [9,13,14] all of which contribute to the occurrence and transformation of Oral potentially malignant disorders (OPMDs) to OSCC [11]. Also, TOP2A was overexpressed in various cancer malignancies including breast, ovarian, pancreatic and prostate [15–19], and it associated to poor prognosis and advanced pathological stages in most cancer types [20]. However, the molecular mechanism and the impact of TOP2A inhibition on the progression of OSCC has not been fully elucidated.

Survivin is highly expressed in most cancers and its overexpression was associated with the inhibition of apoptosis, abnormal proliferation and recent evidence indicated that survivin may be responsible for chemoresistance among various cancer types [21–23]. Likewise, interleukin 6 (IL-6) also involved in the regulation of various pathological condition including cancer [24,25]. Amongst various secretory factors, IL-6 was found to be associated with resistance towards chemo- and radiotherapy, besides promoting tumor growth [25]. Also, it is one of the major cytokines in the tumor microenvironment and Its overexpression has been reported in almost all types of tumors [25]. On the other hand, cancer stem cells (CSCs) are slow dividing cells which can self-renew and are highly drug resistant and thus it contributes to recurrence and metastasis of various tumors [26,27]. Hence, it is important to inhibit the genesis and maintenance of CSCs by targeting transcription factor such as Sox2 and Oct-4 which are especially important for the self-renewal of CSCs [27]. Thus, we investigated the effects of TOP2A inhibition on OSCC and this could lead to a clinically effective strategy to treat OSCC in the future.

## 2. Materials and Methods

### 2.1. Cell Lines and Reagents

Oral squamous cell carcinoma cell line SCC-25 (CRL-1628^™^) was purchased from ATCC^®^ and cultured in DMEM-F12 medium supplemented with 10% fetal bovine serum (FBS), 1.2 g/L sodium bicarbonate, 2.5 mM L-glutamine, 15 mM HEPES and 0.5 mM sodium pyruvate, 400 ng/ml hydrocortisone and 1% antibiotic-antimycotic solution (Corning, Manassas, VA). Cells were passaged at 85–90% confluency and maintained in a humidified air/CO_2_ (19:1) incubator at 37 °C and the media was changed every 3 days. We used the following antibodies for TOP2A (Abcam # ab52934, 1:1000), Survivin (Cell signaling # 2808, 1:1000), IL-6 (Santa Cruz # sc-130326, 1:500), Oct-4 (Cell signaling # 4286, 1:1000), Sox2 (Cell signaling # 23064, 1:1000) and β-Actin (Cell Signaling # 3700, 1:1000). RIPA cell lysis buffer (Thermo Scientific, # 89900), protease inhibitor (Thermo Scientific, # A32965) and phosphatase inhibitor (Sigma-Aldrich # P5726). The ECL Western Blotting detection substrate (Amersham # 2106) was used for protein detection. All other chemicals were of research grade. Etoposide (MedChem Express, # HY-13629) was reconstituted in DMSO for treating the cells.

### 2.2. Western Blotting Analysis

SCC-25 cells were treated with 5μM etoposide (MedChem Express, NJ, USA) and the control groups were treated with 0.1% DMSO for 48h. Cell lysates from control (DMSO) and etoposide treatment groups were prepared using RIPA cell lysis buffer (Thermo Scientific, MA, USA) containing protease inhibitor cocktail (Thermo Scientific, IL, USA). Equal concentrations of protein from control and experimental groups were separated using 4–20% Mini-PROTEAN^®^ TGX^™^ Precast polyacrylamide gels using Trans-Blot Turbo (Bio-Rad, CA, USA). After transferring the protein, the membranes were blocked with 5% non-fat dry milk solution for 1h and the membranes were probed with the following antibodies: anti-TOP2A, anti-Survivin, anti-IL-6, anti-Sox2 and anti-Oct-4 for overnight incubation at +4° C. Then the membranes were incubated with corresponding HRP-conjugated secondary antibody and the protein bands were visualized using the ECL chemiluminescence substrate (Amersham, MA, USA). β-Actin (Sigma-Aldrich, MO, USA) band intensity was used to normalize the protein expression. The protein intensity was quantified using ImageJ software (NIH Image, Bethesda, MD).

### 2.3. Seahorse Metabolic Stress Assay

SCC-25 cells were plated at a density of 2 × 10^4^ cells per well in Seahorse XFe96-well plate. After overnight incubation, cells were treated with 5μM etoposide for 48h. After the treatment period, mitochondrial respiration (Oxygen Consumption Rate, OCR) and glycolytic function (Extracellular Acidification Rate, ECAR) were measured using the XFe96 Seahorse Analyzer (Agilent Technologies, CA, USA) following standardized protocol. Initially, the culture media in the 96 well plate was replaced with Seahorse XF basal medium (Agilent Technologies, CA, USA, # 102353–100) supplemented with 1 mM sodium pyruvate, 2 mM L-glutamine and 25 mM Glucose for mitostress test. For Glycolytic assay, XF base medium was supplemented with only L-glutamine. Then, the 96 well plate was incubated at 37 °C in a CO_2_-free incubator for 1h. The OCR and the ECAR were measured over 100 min with the following compounds injected every three cycles. For mitochondrial respiration assay: oligomycin (3 μM), FCCP (0.25 μM), rotenone (1 μM), and antimycin A (1 μM) (MedChem Express LLC, NJ, USA) as final concentrations in the wells. For the glycolysis assay: glucose (10 mM), oligomycin (3 μM), FCCP (0.25 μM), 2-deoxy-d-glucose (100 mM) (Sigma-Aldrich, St. Louis, USA) were injected sequentially. The results were normalized using the Sulforhodamine B assay to the cell number following the manufacturer’s instructions (Abcam, MA, USA). The final reports were generated, and the results were analyzed using the Wave 2.6.1 software (Agilent Technologies, Santa Clara, CA).

### 2.4. TCGA Gene Expression Profile

The TOP2A gene expression data was extracted from the publicly available data set cBioPortal, The Cancer Genome Atlas Studies, (TCGA). The mRNA expression of the indicated cancer samples was assessed as Z scores relative to all samples and the quantification was based on the RNA-Seq by Expectation-Maximization (RSEM). The correlation between the TOP2A and the metastasis stages were assessed per the American Joint Committee on Cancer Metastasis Stage Code. The representative H&E staining from Head and Neck cancer patients with high and low TOP2A expression (TCGA-QK-A6IJ and TCGA-BB-7866) were assessed from cBioPortal data base.

### 2.5. Statistical analysis

Statistical analysis was performed in GraphPad Prism, version 8 (GraphPad Software Inc., San Diego, CA). Significant differences were analyzed using One-way ANOVA. Multiple comparisons between treatment and control groups were performed using Dunnett’s test. A value of *P* less than 0.05 was considered statistically significant.

## 3. Results

### 3.1. Effects of etoposide on TOP2A protein expression on oral cancer cells

**Figure 1:**
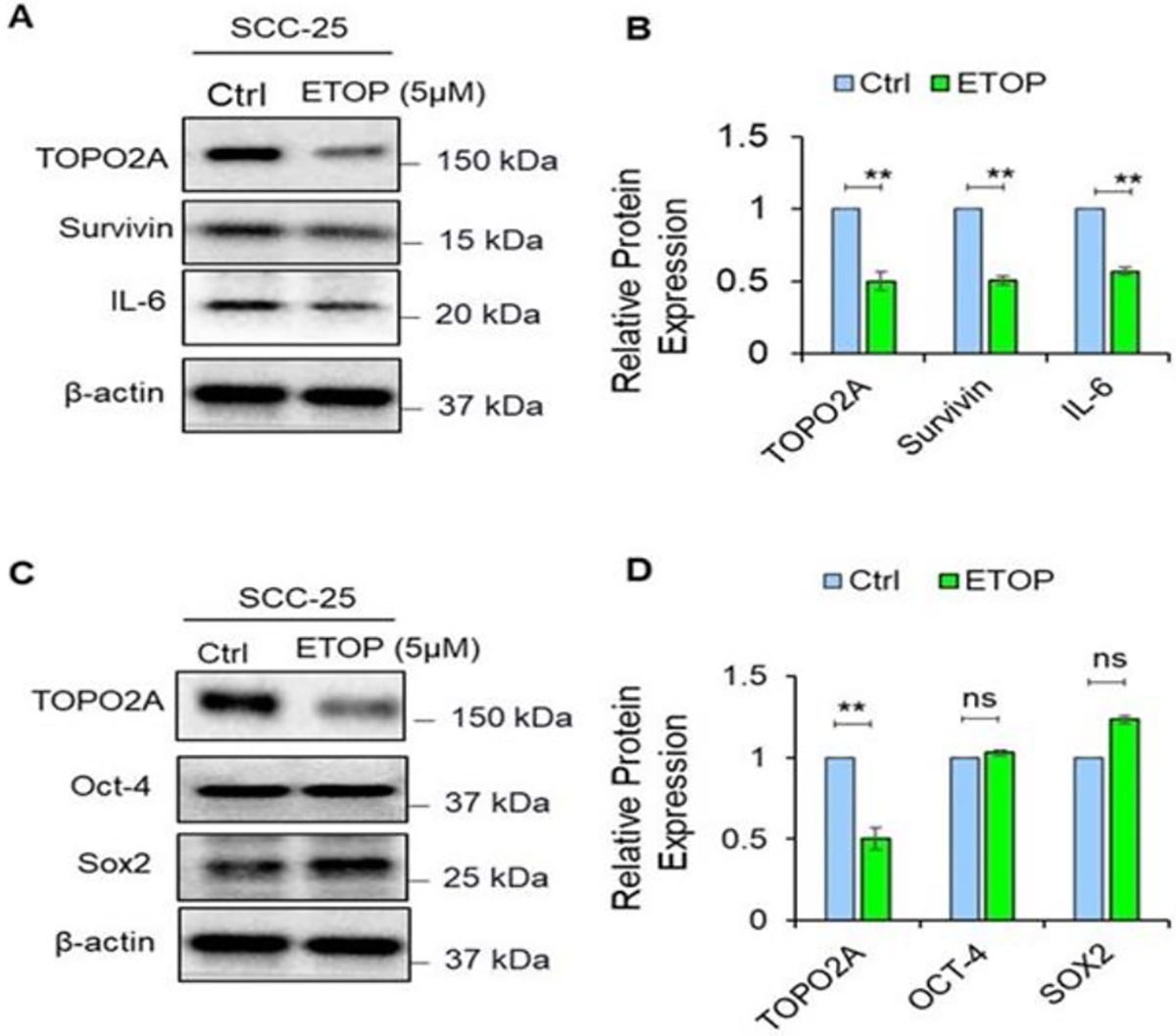
Effect of TOP2A inhibition on oral cancer cell progression marker. (A, B) Expression of TOP2A, Survivin and IL-6 were analyzed from proteins extracted from control and etoposide treated groups were analyzed by western blotting. The protein extracts (25 μg of protein) were separated by 4–20% Mini-PROTEAN^®^ TGX^™^ Precast Protein Gels. After electro-blotting, the separated proteins were probed with the corresponding antibody. Significant alteration in the expression pattern of the indicated proteins was observed in treatment groups as compared to untreated control tumor. β-actin was used as a loading control. (C, D) Expression of TOP2A and the cancer stem cell marker (OCT-4 and Sox2 were analyzed as mentioned above. The band intensity between control and treatment groups were quantified using Image J. (**P≤0.01), ns - non-significant.

Etoposide is a topoisomerase II inhibitor and an anticancer medication used in combination with other chemotherapy medications to treat testicular cancer or small cell lung cancer [19,28]. Thus, we tested the effects of etoposide on TOP2A protein expression in SCC-25 oral cancer cell line after 48h treatment. Etoposide treatment resulted in a significant reduction of TOP2A protein expression in oral cancer cell line (Figure 1A and B).

### 3.2. Effects of TOP2A inhibition on cancer progression and survival signaling proteins

Survivin, an inhibitor of apoptosis protein, is highly expressed in most cancers and associated with chemotherapy resistance, increased tumor recurrence, and shorter patient survival [29,30]. Therefore, we sought to see the effects of TOP2A inhibition on survivin protein. As expected, the expression of survivin was significantly higher in OSCC cells. However, etoposide, a TOP2A inhibitor significantly inhibited the protein expression of survivin in OSCC cells (Figure 1A and B). Furthermore, we sought to analyze the effect of TOP2A inhibition on IL-6 protein expression, which is one of the key cytokines in the tumor microenvironment confer resistance to chemo and radiotherapy and its overexpression has been reported in almost all types of tumors. Strikingly, etoposide significantly reduced the protein expression of IL-6 in OSCC cells (Figure 1A and B).

### 3.3. Impact of TOP2A inhibition on Cancer stem cell self-renewal marker in OSCC Cells

Cancer stem cell self-renewal function is a critical characteristics of tumor cells for its initiation, proliferation, and therapy resistance [21,31]. Thus, we analyzed the effects of TOP2A inhibition by etoposide on cancer stem cell self-renewal marker Oct-4 and Sox2. Our results indicate that inhibition of TOP2A has no significant impact on Oct-4 and Sox2 protein expression (Figure 1C and D). This indicates that, the TOP2A may not regulate the cancer stem cell self-renewal function directly by regulating through Oct-4 and Sox2 rather it regulates by other mechanism.

### 3.4. Impact of TOP2A inhibition on Mitochondrial Respiration of OSCC Cells

**Figure 2:**
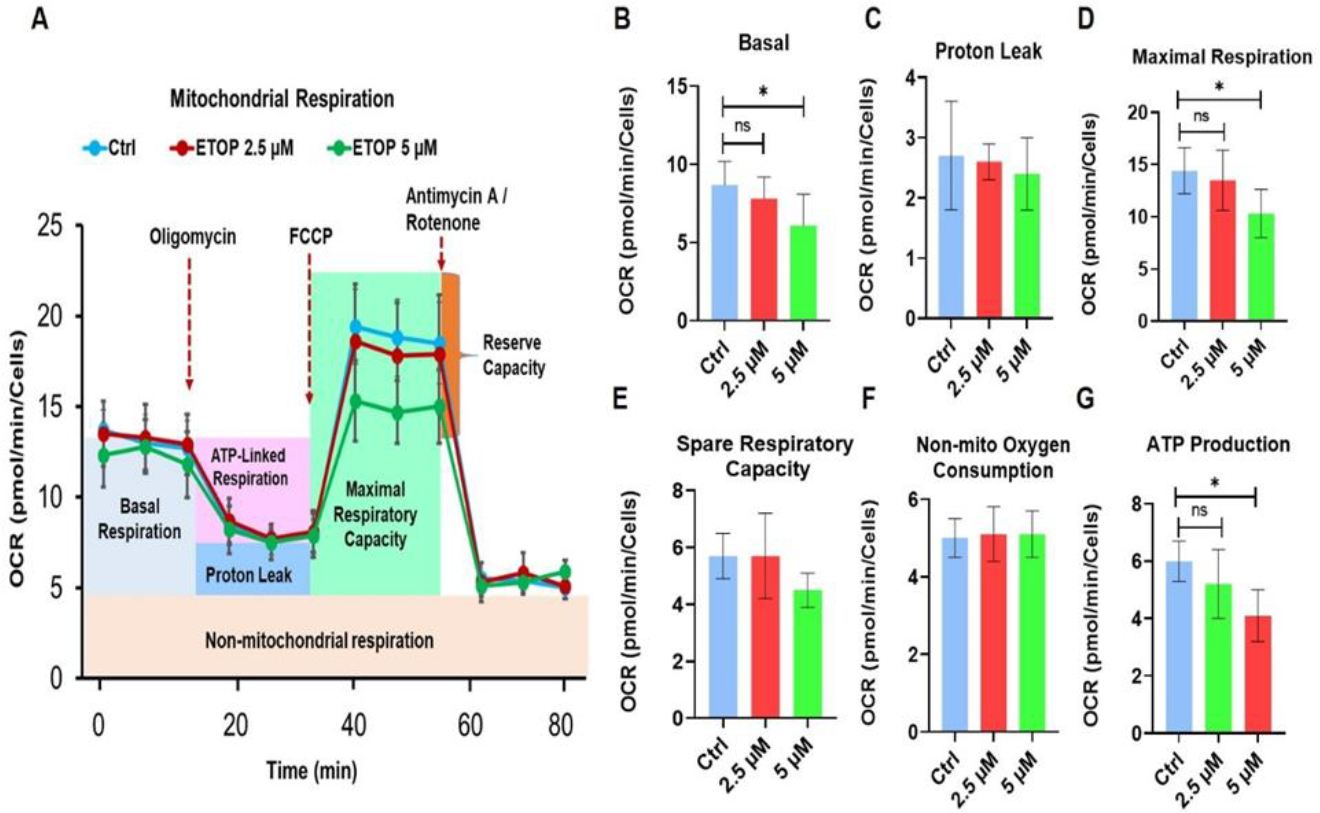
Effects of TOP2A inhibition on mitochondrial respiration. (A-C) SCC-25 cells (2×10^4^) were seeded in a seahorse XFe96 microplate. Cells were treated with 5μM etoposide for 48 hours. For mitostress assay, Oxygen consumption rate (OCR) was determination in cells incubated in XF base medium supplemented with 10 mM glucose, 1 mM sodium pyruvate, and 2 mM L-glutamine. Then cells were equilibrated in non-CO2 incubator for 1 h. During the incubation, mitochondrial inhibitors oligomycin (1 μM), FCCP (0.5 μM), and rotenone/antimycin A (0.5 μM) were injected at the XFe96 sensor cartridge. Normalization of cell numbers was performed using SRB assay. (*P≤0.05), ns-non-significant. The results were analyzed using the Wave 2.6.1 software and the reports were generated using Mito Stress Test Report Generator.

Metabolic adaptation of cancer cells through reprogramming is one of the hallmarks of tumor for their bioenergetic and biosynthetic demand for cell proliferation and survival [32]. Thus, we analyzed the effect of TOP2A inhibition on oral cancer cell metabolism by assessing mitochondrial respiration of OSCC cells using Mito Stress test (Figure 2A). Our results indicate that the basal OCR rate was significantly inhibited in SCC-25 cells treated with etoposide (Figure 2B). In addition, the maximum respiration was significantly inhibited etoposide (Figure 2D). Strikingly, the important mitochondrial parameter ATP production rate was also significantly reduced in etoposide treated oral cancer cells (Figure 2D). However, no significant differences were observed in proton leak, spare respiratory capacity and non-mitochondrial consumption (Figure 2C, E, F).

### 3.5. Effects of TOP2A inhibition on Glycolytic Function on OSCC Cells

**Figure 3:**
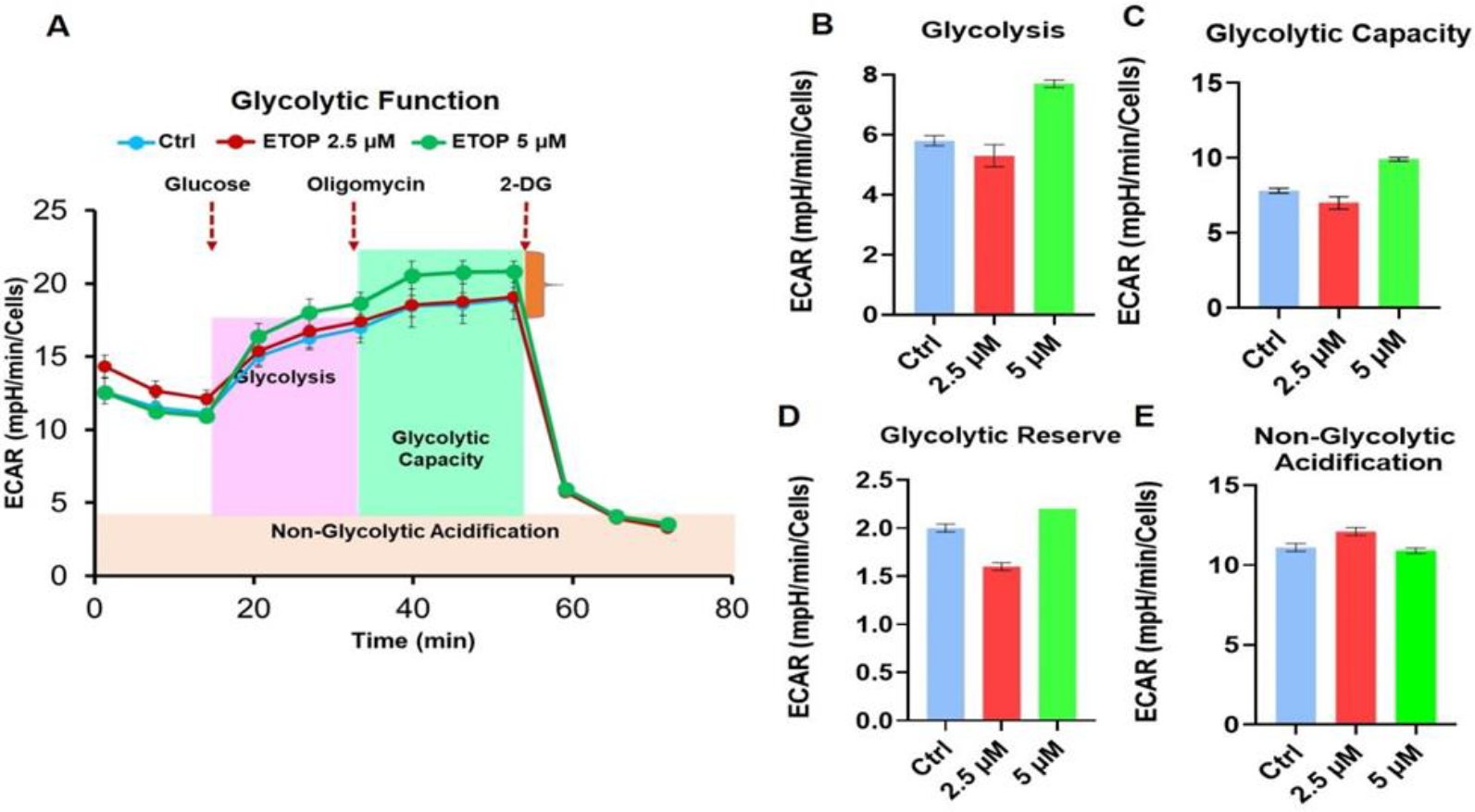
Effects of TOP2A inhibition on glycolytic function. (A-C) SCC-25 cells (2 × 10^4^ per well) were plated in a seahorse XFe96 microplate and the cells were treated with 5 μM etoposide for 48 hours. The compounds including 2-DG were used to inhibit glycolysis to acquire a baseline extracellular acidification rate (ECAR). The following parameters were assessed: (B) glycolysis (C) glycolytic capacity (D) glycolytic reserve and (non-glycolytic acidification). Normalization of cell numbers was performed using SRB assay following manufacturer’s instruction. The results were analyzed using the Wave 2.6.1 software and the reports were generated using Mito Stress Test Report Generator.

Evidences indicate that glycolysis is indeed sufficient to fuel the rapid energy required for cell proliferation and increased glycolysis is considered as a hallmark of cancer [32]. In addition, cancer cells easily adapt to intrinsic or extrinsic cues from the microenvironment through the metabolic plasticity [32]. Therefore, we intend to assess the effect of etoposide on the glycolytic function of OSCC cells. The results demonstrated that the etoposide has no impact on the glycolytic functional parameters, including glycolysis (Figure 3 and B), glycolytic capacity (Figure 3C), glycolytic reserve (Figure 3D) and non-glycolytic acidification (Figure 3E) in SCC-25 cells. These results confirm that etoposide impacts the cancer cells by affecting the mitochondrial respiration rather than glycolytic function.

### 3.6. TOP2A overexpression associated with progression and metastasis of oral cancer

**Figure 4:**
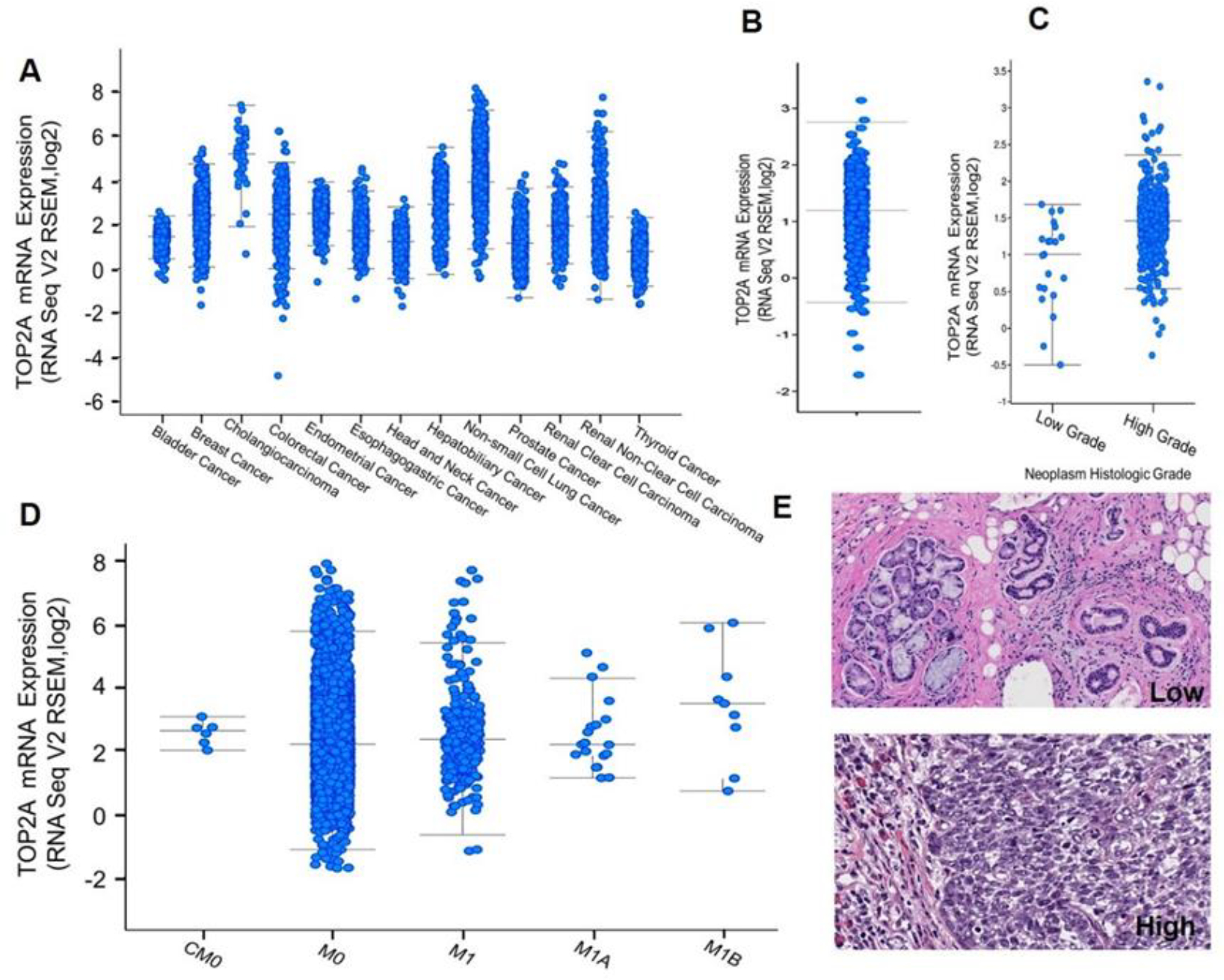
High *TOP2A* expression is associated with cancer metastasis and poor prognosis. (A) *TOP2A* mRNA expression among various cancer types including Head and Neck Squamous cell carcinoma. (B) Differential mRNA expression level of *TOP2A* in Head and Neck carcinoma patients. (C) *TOP2A* mRNA expression was associated with histologic grade of tumor. (D) Association of *TOP2A* mRNA with the metastatic stages of cancer (American Joint Committee on Cancer Metastasis Stage Code). (E) *TOP2A* mRNA expression levels (low and high) correlation with H & E images Head and Neck squamous cell carcinoma. These data were generated based on the publicly available database, cBioPortal for Cancer Genomics.

Analysis of TCGA RNA-seq data from cBioPortal cancer genomic database showed that *TOP2A* mRNA expression was significantly higher in most cancer types including Head and Neck cancer (Figure 4A). In addition, we examined the publicly available data sets of patient samples and found the elevated expression of *TOP2A* was correlated significantly with disease stage (Figure 4B and D) and also with the metastatic index (Figure 4D). Moreover, the H and E images of oral biopsies with epithelial changes were correlated with *TOP2A* expression (Figure 4E).

**Figure 5:**
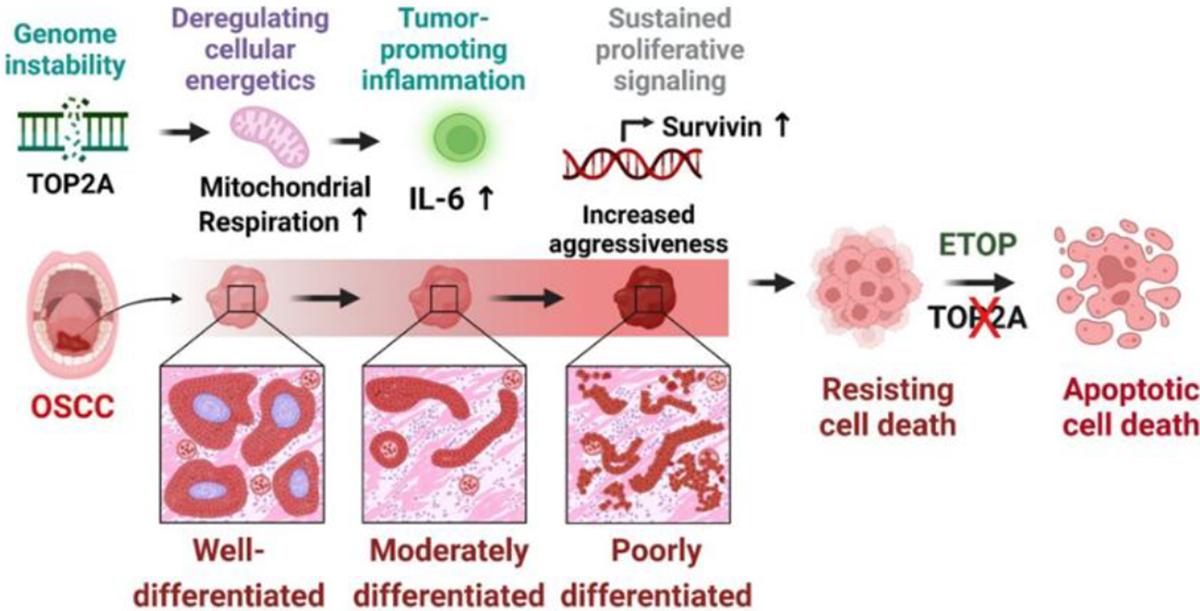
Illustration on the oncogenic role of TOP2A in OSCC cells. The overexpression of TOP2A associated with poorly differentiated OSCC with increased aggressiveness through deregulated cellular energetics by enhanced mitochondrial respiration, increased expression of tumor promoting inflammatory marker (IL-6) and sustained proliferation signals (Survivin). Treatment of OSCC cells with TOP2A inhibitor etoposide abrogated these processes and induces apoptotic cell death of OSCC cells.

Collectively, we illustrated the oncogenic effect of TOP2A in OSCC cells, the overexpression of TOP2A was associated with the poorly differentiated OSCC with increased aggressiveness with enhanced mitochondrial respiration, increased tumor promoting inflammatory signaling with sustained proliferation signals. Treatment of OSCC cells with TOP2A inhibitor etoposide abrogated these processes and induces apoptotic cell death of OSCC cells (Figure 5).

## 4. Discussion

OSCC accounts for approximately 90% of all oral cancers. Moreover, according to the Global Cancer Observatory (GCO), by 2040, the overall incidence of OSCC will rise by 40% with a subsequent increase in the mortality rate [11]. Despite various comprehensive therapies currently available, there are still several contributing factors that may impact the prognosis of OSCC. TOP2A, a member of DNA topoisomerase has been involved in the development and progression of multiple tumors associated with the increased cell proliferation and tumor grade [9,19,20,33,34]. The inhibition of TOP2A has been associated with reduced tumorigenesis of various cancers [17,35]. However, the effects of TOP2A inhibition on OSCC pathogenesis in less characterized. Recent results from our laboratory confirmed the inhibition of TOP2A by etoposide. The IC50 for etoposide in SCC-25 cell line was previously determined to be 5μM [36], the same concentration used in our experiments. Firstly, we confirmed that etoposide significantly inhibited the protein expression of TOP2A in SCC-25 oral cancer cell line. As a result of TOP2A inhibition, etoposide significantly inhibits the anti-apoptotic protein survivin and IL-6. Conversely, we extended to analyze some of the cancer stem cell self-renewal marker Sox2 and Oct-4. Yet, our results showed that inhibition of TOP2A was not associated with any significant impact on these proteins. Moreover, metabolic reprogramming was significantly inhibited which was evidenced by inhibition of basal respiration, spare respiratory capacity, and ATP production. The glycolytic function was not impacted by etoposide.

Previous studies clearly indicated that the activation of TOP2A was associated with cell proliferation, chemotherapy resistance, and disease aggressiveness of variety of human cancers [15,16,18,33,37,38]. However, the inhibition of TOP2A dramatically decrease the cellular proliferation of various cancer cells [14,17,35,37]. Thus, we investigated to see the effect of TOP2A inhibition on cell survival and apoptotic effects. The abnormal expression of anti-apoptotic proteins resulting in less effective or ineffective antitumor treatments and the inhibition of anti-apoptotic or reactivation of cell apoptosis becomes a key approach to overcome anticancer therapy resistance [39,40]. Survivin is a member of the Inhibitor of Apoptosis (IAP) family and its expression has been previously shown to be associated with tumor cell division and proliferation. In addition, survivin orchestrates tumor cell survival by regulating enhanced cell proliferation, migration, and invasion [28,41,42].

Our results indicate that inhibition of TOP2A significantly decreased the expression level of survivin in OSCC cells. Thus, decreased level of survivin by TOP2A inhibition may affect its physically interaction with caspases-3, -7, and -9 and enhance pro-apoptotic effects, indicating that survivin also is a caspase inhibitor [40]. Also, it inhibits cell death by interfering with caspase-9 processing, the main inhibitor in intrinsic pathway of apoptosis [40]. In addition, survivin interact with IAP proteins to form a heterocomplex with enhanced stability against ubiquitination and proteasomal degradation [39]. Furthermore, evidence indicates that survivin involved in cell cycle process by localizes to the mitotic spindle and plays an important role in regulating mitosis by interacting with tubulin [29]. Moreover, survivin can be phosphorylated by CDK1 a key kinase for cell cycle regulation [30].

A strong association has been reported between increased levels of interleukin-6 (L-6) in the tumor microenvironment, a major cytokine, and cancer. Tumorigenesis is enhanced though IL-6 mediated regulation of multiple signaling pathways involved in apoptosis, proliferation, angiogenesis, invasiveness, metastasis, and, most importantly, metabolism [24,43,44]. Regardless of various therapeutic options, certain secretory factors released by the tumor cells into the microenvironment have been found to confer resistance towards chemo- and radiotherapy, besides promoting growth [25,45,46] In the present study, etoposide significantly reduced the protein expression of IL-6. This result is consistent with the recent finding, where IL-6 promotes OSCC progression via JAK/2/STAT3/SOX/4/NLRP3 signaling [47]. However, silencing of IL-6 resulted in a significant inhibition of head and neck squamous cell proliferation and cell migration [48]. Furthermore, increased levels of IL-6 suppressed radiation-induced cell death and blockade of IL-6 signaling by tocilizumab sensitized OSCC tumor cells to radiation [49].

We also sought to evaluate the effect of TOP2A inhibition on the markers which are involved in the CSCs, self-renewal ability and multilineage differentiation potential that are a critical for tumor cell initiation, growth, and resistance to therapy [27]. Our results demonstrated that etoposide has no effect on the cancer stem cell self-renewal marker Sox2 and oct-4. However, it may have impact on other markers or transcription factors such as KLF4, C-MYC, Nanog and SALL4 [31,50–52]. Metabolic reprogramming is one of the important hallmarks of tumors and it is associated with resistance of tumor cells to antitumor drugs [32] In addition, actively growing tumor cells exhibit increased mitochondrial biogenesis and respiration for ATP generation to meet energy demands and maintain survival [53,54]. Therefore, we evaluated the impacts of TOP2A inhibition on mitochondrial respiration and glycolytic function of OSCC cells. Our results indicate that TOP2A inhibition by etoposide was found to be effective on the mitochondrial respiration by reducing the basal, maximal respiration, with decrease in ATP production in OSCC cells. However, the glycolytic function was not impacted by etoposide.

## 5. Conclusion

Our preliminary results clearly indicate that TOP2A inhibition by etoposide decreases the antiapoptotic proteins and mitochondrial respiration of OSCC cells. Further in-depth investigation is required to better understand the molecular mechanisms of TOP2A regulation on OSCC.

## Data Availability

The data generated as a part of the study has been submitted in this article.
